# Alterations in co-abundant bacteriome in colorectal cancer and its persistence after surgery: a pilot study

**DOI:** 10.1038/s41598-022-14203-z

**Published:** 2022-06-14

**Authors:** Chin-Wen Png, Yong-Kang Chua, Jia-Hao Law, Yongliang Zhang, Ker-Kan Tan

**Affiliations:** 1 Department of Microbiology and Immunology, Yong Loo Lin School of Medicine, National University of Singapore, Singapore, Singapore; 2 Division of Colorectal Surgery, Department of Surgery, National University Hospital, Singapore, Singapore; 3 Department of Surgery, Yong Loo Lin School of Medicine, National University of Singapore, Singapore, Singapore

**Keywords:** Cancer, Microbiology, Medical research

## Abstract

There is growing interest in the role of gut microbiome in colorectal cancer (CRC), ranging from screening to disease recurrence. Our study aims to identify microbial markers characteristic of CRC and to examine if changes in bacteriome persist after surgery. Forty-nine fecal samples from 25 non-cancer (NC) individuals and 12 CRC patients, before and 6-months after surgery, were collected for analysis by bacterial 16S rRNA gene sequencing. Bacterial richness and diversity were reduced, while pro-carcinogenic bacteria such as *Bacteroides fragilis* and *Odoribacter splanchnicus* were increased in CRC patients compared to NC group. These differences were no longer observed after surgery. Comparison between pre-op and post-op CRC showed increased abundance of probiotic bacteria after surgery. Concomitantly, bacteria associated with CRC progression were observed to have increased after surgery, implying persistent dysbiosis. In addition, functional pathway predictions based on the bacterial 16S rRNA gene data showed that various pathways were differentially enriched in CRC compared to NC. Microbiome signatures characteristic of CRC comprise altered bacterial composition. Elements of these dysbiotic signatures persists even after surgery, suggesting possible field-change in remnant non-diseased colon. Future studies should involve a larger sample size with microbiome data collected at multiple time points after surgery to examine if these dysbiotic patterns truly persist and also correlate with disease outcomes.

## Introduction

The human microbiome is a second genome of the human body where microorganisms establish a symbiotic relationship with the host in complementing metabolic deficits, protection from invading pathogens and maintaining immune homeostasis^1^. There is increasing evidence showing that gut microbiota influenced CRC development through the virulence factors of pathogenic bacterial strains, and recent reviews and papers have suggested a close correlation between certain microbial strains and CRC^2–6^. Bacteria such as *Bacteroides fragilis*, *Fusobacterium nucleatum*, and *Escherichia coli* have been identified to be enriched in patients with CRC^7,8^. Various molecular events as well as changes to the tumor microenvironment which are related to the enrichment of specific bacterial species were found to promote CRC tumorigenesis in various animal and *ex-vivo* models^9–11^. These specific microbial strains associated with CRC provided promising opportunities to develop diagnostic tools or treatment biomarkers for CRC.

However, the relationship between the bacteriome and CRC appear complex as there is unlikely a single pathogenic bacteria that is related to CRC, unlike the direct causal relationship that is established between *Helicobacter pylori* and gastric cancer^12^. Different microbial strains that are associated with CRC have been reported, some of which were not reproducible in other studies^13^. For example, Dejea et al. found that there was no consistent bacterial genus associated with tumors by high-throughput sequencing in 30 CRC and 6 adenoma human samples^13^. In another study, the widely studied *Fusobacterium* that is increasingly associated with CRC was only found to be elevated in 20–30% of CRC patients and was not a consistent finding^14^. On the other hand, other studies found differing microbes that triggered neoplasia and also proposed associated mechanisms. For example, *Bacteroides fragilis* was found to promote Th17 development which limits the availability of IL2 in the local microenvironment, while *Escherichia coli* releases colibactin which is a genomic product of polyketide synthase island that is carcinogenic and promotes CRC development^6,15^.

A common finding in all studies was the alterations or imbalance in gut microbial composition unique to CRC patients, which was otherwise known as microbial dysbiosis. As Katarzyna et al. highlighted, microbial “dysbiosis” is a loosely defined term that can refer to microbial compositional changes, imbalance, or alterations in specific microbial lineages. It can refer to taxonomic or functional dysbiosis^16^. Dysbiosis may not be adequate to the task of establishing microbiota causality in disease as such causal relationships need to be made on firm scientific basis and fulfill the “commensal Koch’s postulates”^17,18^. However, existing literature suggests that the initiation and development of CRC appear to be fueled by a collective microbial dysbiosis with interactions amongst numerous microbes, rather than propelled by specific pathogenic microbes.

It is also inconclusive if the state of microbial dysbiosis associated with CRC continues to persist after curative surgery. Few studies have investigated the microbial alterations after curative CRC surgery. Kong et al. reported a reduced ratio of *Bacteroidetes/Firmicutes* after surgery, which could contribute to intestinal inflammation^19^. Tumor-associated microbes including *Enterococcus* and *Fusobacterium* were also found to be reduced. However, beneficial obligate anaerobes such as *Prevotella* were also reduced postoperatively. It is postulated that if the microbial dysbiosis is associated with the cancer, then resection of the tumour should not only remove the lesion but also reverse the dysbiosis that accompanied CRC. On the other hand, persistent dysbiosis after surgery may suggest possible field change in the “non-diseased” colon that predisposed the patient to CRC in the first place.

In this study, we thus seek to examine alterations in co-abundant bacterial strains in CRC patients before (pre-op) and after (post-op) surgery and to also compare them to non-cancer (NC) individuals. Using 16S rRNA gene sequencing, we first performed the classical community analysis and statistical tests to compare the gut microbial community structure and composition between CRC patients before and after surgery and NC individuals. We then applied functional network analysis to further examine the differences among them.

## Results

### Study population

In this study, we have included a total of 37 individuals, of which 12 were CRC patients and 25 were NC controls. Using T statistics (non-centrality parameter) power calculation, the number of samples included in this study provide sufficient power to detect effect size of 2, at 95% confidence with 80% power^26^. The participant and clinical data are summarized in Tables 1 and 2, respectively. Bacterial 16S rRNA gene sequencing of 49 fecal samples from CRC patients and NC controls were performed to investigate the compositional changes of the microbiome in CRC patients pre- and post-operatively (pre- and post-op). A total of 2,559,498 filtered sequences were obtained with an average of 52,234 sequences per sample (range 3531–98,096). A rarefaction curve for richness is plotted to analyse the sequencing depth of the samples (Supplementary Fig. 1).

**Table 1:** Overview of participant clinical characteristics.

	Cancer (% or SD) n = 12	Healthy (% or SD) n = 25
Age, mean	63.8 (± 9.3)	61.6 (± 8.9)
**Gender**		
Male	7 (58.3%)	14 (56.0%)
Female	5 (41.7%)	11 (44.0%)
Body mass index, mean	25.8 (± 4.0)	25.2 (± 4.4)
Adjuvant chemotherapy	7 (58.3%)	–

**Table 2:** Clinical data of cancer group patients.

Subject	Age	Gender	Race	Tumor location	TNM stage	Surgery
M02-P	57	Male	Malay	Sigmoid	3	Anterior resection
M03-P	74	Female	Chinese	Descending	3	Left hemicolectomy
M04-P	56	Female	Malay	Sigmoid	1	Anterior resection
M06-P	60	Female	Malay	Sigmoid	3	Anterior resection
M07-P	67	Male	Chinese	Sigmoid	2	Anterior resection
M08-P	46	Male	Chinese	Rectum	3	Anterior resection
M09-P	59	Female	Indian	Rectum	2	Anterior resection
M11-P	65	Male	Chinese	Sigmoid	2	Anterior resection
M12-P	65	Male	Indian	Rectosigmoid	1	Anterior resection
M13-P	69	Male	Chinese	Sigmoid	3	Anterior resection
M15-P	82	Female	Chinese	Rectosigmoid	3	Anterior resection
M16-P	66	Male	Chinese	Sigmoid	1	Anterior resection

### Bacterial diversity is altered in CRC patients compared to NC individuals

**Figure 1:**
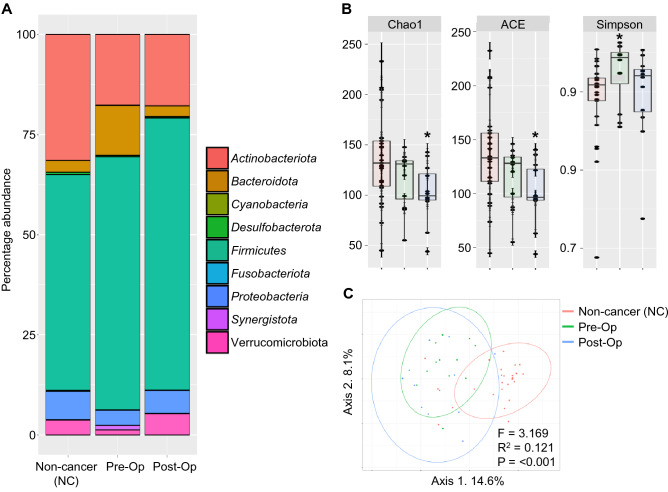
Fecal bacterial compositions are different between NC and CRC patients. (**A**) Bar chart shows the top bacterial phyla present in all samples. (**B**) Comparisons of bacterial alpha diversity in CRC to NC control group. * denotes p < 0.05 (Wilcoxon rank sum test). (**C**) Principal coordinates analysis (PCoA) of bacterial beta diversity derived from Bray–Curtis distances among specimens is shown. F statistics, R^2^ & p values of the comparison are presented (PERMANOVA).

*Firmicutes, Actinobacteria, Verrucomicrobia and Bacteroidetes* are amongst the most abundant bacteria phyla found in all samples (Fig. 1A), Global microbial alpha diversity was assessed using Chao1, ACE and Simpson across 3 groups. Chao1 and ACE indices were significantly decreased in post-op patients (p < 0.05). In addition, an increase in Simpson index was observed for pre-op patients (p < 0.05) when compared to other groups in the analysis (Wilcoxon rank sum test, Fig. 1B). Using principal coordinate analysis (PCoA) of the Bray–Curtis dissimilarity distances, beta diversity analysis of the groups showed that there is a clear segregation between CRC patients and NC (Fig. 1C). Furthermore, microbial composition of post-op CRC patients presented a more scattered distribution, indicating a different microbial composition as compared to pre-op CRC patients and NC. In all, the data suggest reduced fecal bacterial species richness and diversity in the CRC patients compared to the NC.

### Taxonomic differences in pre- and post-operative CRC patients compared to NC controls

**Figure 2:**
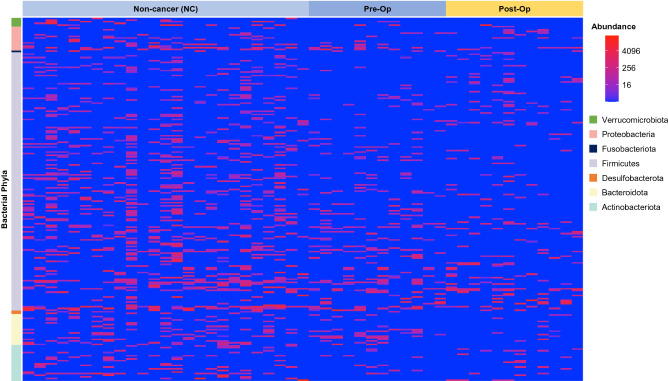
Heatmap showing differentially abundant bacterial phyla across NC and CRC patients. Top differentially abundant bacterial phyla in CRC compared to NC control are shown (ɑ < 0.01, Wald test).

An overview of differentially abundant bacteria in CRC patients and NC fecal specimens was shown in Fig. 2. Based on the heatmap representation of bacterial abundance, it is clear that alterations in the abundance of amplicon sequence variants (ASVs) belonging to *Actinobacteria, Firmicutes*, *Proteobacteria, Verrucomicrobia and Bacteroidetes* phyla across the different groups were present. Of note, several specific ASVs belonging to *Actinobacteria* and *Firmicutes* were increased, whereas some *Bacteroidetes* ASVs were reduced in post-op CRC patients compared to pre-op patients.

**Figure 3:**
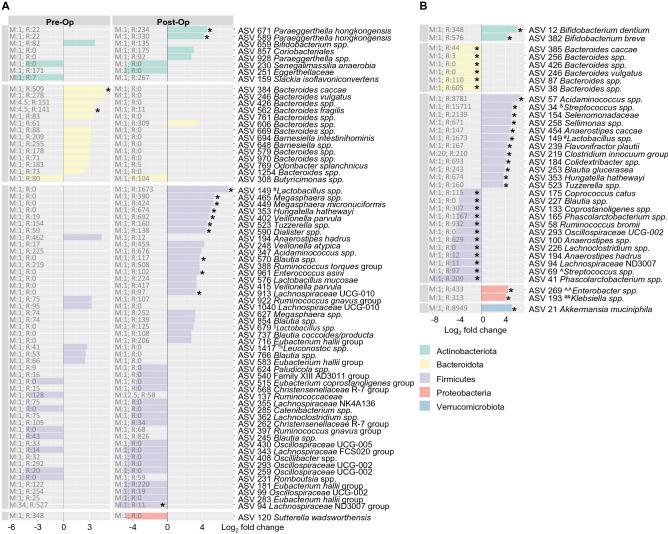
Abundance of specific bacterial ASVs are different in CRC patients compared to NC control and changes after surgery. (**A**) Bar chart shows bacterial ASVs that are significantly different in CRC patient pre- and post-op compared to NC controls (ɑ ≤ 0.0001, *denotes Benjamini–Hochberg adj < 0.0001). “M;R” denotes median and range of Deseq2 normalised counts in pre-op & post-op groups. (**B**) Bar chart shows bacterial ASVs that are significantly different in CRC post-op compared to pre-op (Paired test, ɑ ≤ 0.0001, *denotes Benjamini–Hochberg adj < 0.0001). “M;R” denotes median and range of Deseq2 normalised counts in post-op group. Plots showing detail media & range in NC, pre-op & post-op are summarised in Supplementary Data 1 and 2. Abbreviated ASV annotation as below; ASV149 #*Lactobacillus acidophilus/casei/crispatus/gallinarum*; ASV679 $*Lactobacillus crustorum/farciminis/formosensis/heilongjiangensis/mindensis/musae/nantensis*; ASV1417%*Leuconostoc carnosum/citreum/garlicum/holzapfelii/lactis*; ASV_34 &*Streptococcus alactolyticus/equinus/gallolyticus/macedonicus/pasteuri/pasteurianus*; ASV_69 ^*Streptococcus lutetiensis/salivarius/thermophilus*; ASV_269 ^^*Enterobacter aerogenes/asburiae/bugandensis/cancerogenus/cloacae/hormaechei/ludwigii/mori/roggenkampii/soli/tabaci*; ASV_193 ##*Klebsiella pneumoniae/quasivariicola/variicola.*

Further detailed analysis revealed the level of changes in abundance of specific bacterial ASVs in pre- and post-op CRC patients compared to NC (Fig. 3, Supplementary Data 1). As shown in Fig. 3A, pre-op CRC patients have a higher abundance of several *Bacteroidetes* ASVs. *Bacteroides caccae, Bacteroides vulgatus and Bacteroides fragilis* were found to be increased in pre-op CRC patients (ɑ ≤ 0.0001, Fig. 3A, pre-op column)*.* In addition, other bacteria species including, ASV 769 *Odoribacter splanchnicus* ASV 694 *Barnesiella intestinihominis*, ASV 194 *Anaerostipes hadrus*, ASV 766 *Blautia spp.* and ASV 583 *Eubacterium hallii group* were increased by more than 2 Log_2_ fold in pre-op CRC compared to NC (Fig. 3B)^27–32^. However, these differences were no longer found in post-op CRC compared to NC. Of note, the abundance of *Bacteroides *spp., *B. caccae* and *B. vulgatus* were reduced in post-op CRC compared to pre-op based on paired analysis (Fig. 3B). In contrast, fecal samples from pre-op patients have reduced ASV 308 *Butyricimonas *spp., ASV 515 *Eubacterium coprostanoligenes* group, ASV 285 *Catenibacterium *spp. and ASV 343 *Lachnospiraceae* FCS020 group, and these bacterial groups remained reduced even after post-op. Additionally, paired analysis revealed the influence of surgery on specific bacterial ASVs in CRC patients. These included the increase in ASV 269 *Enterobacter *spp., ASV 193 *Klebsiella spp*. and ASV 21 *Akkermansia muciniphila.*

### Microbial functional features associated with CRC development

#### Overview of pathway analysis between groups

**Figure 4:**
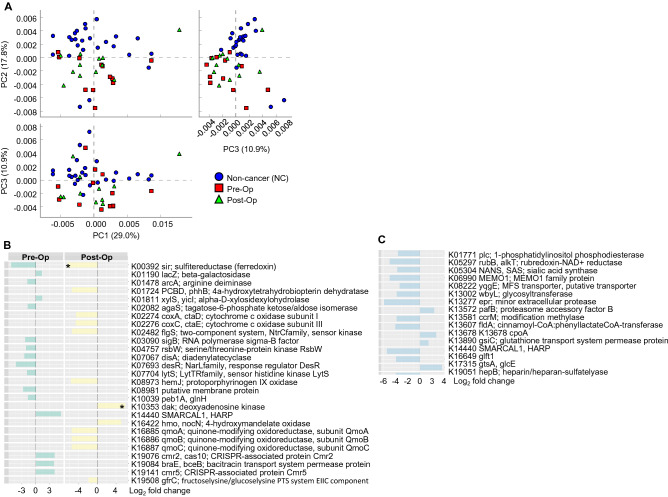
Changes in functional profile of bacterial composition in NC and CRC patients. (**A**) Principal coordinate analysis (PCoA) of PICRUSt2-projected functional profiles (level 3, KEGG orthology (KOs)) between NC, CRC patients pre- and post-op (ANOVA Tukey–Kramer post hoc comparison, Benjamini–Hochberg correction, padj < 0.05). (**B**) Bar chart shows top differentially represented KOs in CRC patients pre- and post-op compared to NC control (ɑ < 0.000001, all comparisons Benjamini–Hochberg adj < 0.001, *p. adj < 0.0001). (**C**) Bar chart shows top differentially represented KOs in CRC post-op compared to pre-op (Paired test, p < 0.001).

Gene content and functional pathways were inferred from the bacterial 16S rRNA gene sequencing data using Picrust2. The top differentially abundant KEGG (KOs) profiles from NC, pre- and post-op groups were compared using ANOVA Tukey–Kramer post-hoc comparison with Benjamini–Hochberg correction^33^. PCoA plot (Fig. 4A, Supplementary Data 2) showed that the KOs profiles from NC are significantly different from both pre- and post-op CRC (Benjamini–Hochberg FDR < 0.05).

There were 66 and 300 differentially abundant KOs identified in pre- and post-op CRC compared to NC control group respectively (BH adjusted P ≤ 0.0015, Supplementary Data 3 and 4). These KOs were mapped to KEGG pathways including metabolic pathways (23% in pre-op, 34% in post-op); biosynthesis of secondary metabolites (10% in both pre-op and post-op) and microbial metabolism in diverse environments (3% in pre-op, 8% in post-op). The differentially abundant KOs were also assigned to BRITE hierarchies. The most represented BRITE hierarchies included; enzymes (51% in pre-op, 34% in post-op), two-component system (6% in pre-op, 3% in post-op), prokaryotic defense system (5% in pre-op, 1% in post-op) and transcription machinery (5% in pre-op, 1% in post-op).

Interestingly, KOs including K00392 sir; sulfite reductase (ferredoxin) was found to be significantly reduced in pre- and post-op (−5.3 and − 6.3 Log_2_ fold change respectively, adj. P ≤ 0.0001) compared to NC (Fig. 4B,C, Supplementary Fig. 2 and Supplementary Data 2), which mapped to the MODULE “M00176 Assimilatory sulfate reduction, sulfate =  > H2S”. The importance of the altered sulfur metabolism was also identified in subsequent detailed pathway enrichment analysis using clusterProfiler R package^34^. Additionally, ABC transporters remain over-represented in post-op (when compared with NC or pre-op), thus suggesting surgery did not change the bacteria group carrying out this function in patients’ gut. Key KOs in ABC transporter include the trehalose/maltose transport system, cellobiose transport system, urea transport system and fluoroquinolone transport system (Supplementary Data 3 and 4).

#### Pre-op CRC vs NC

**Figure 5:**
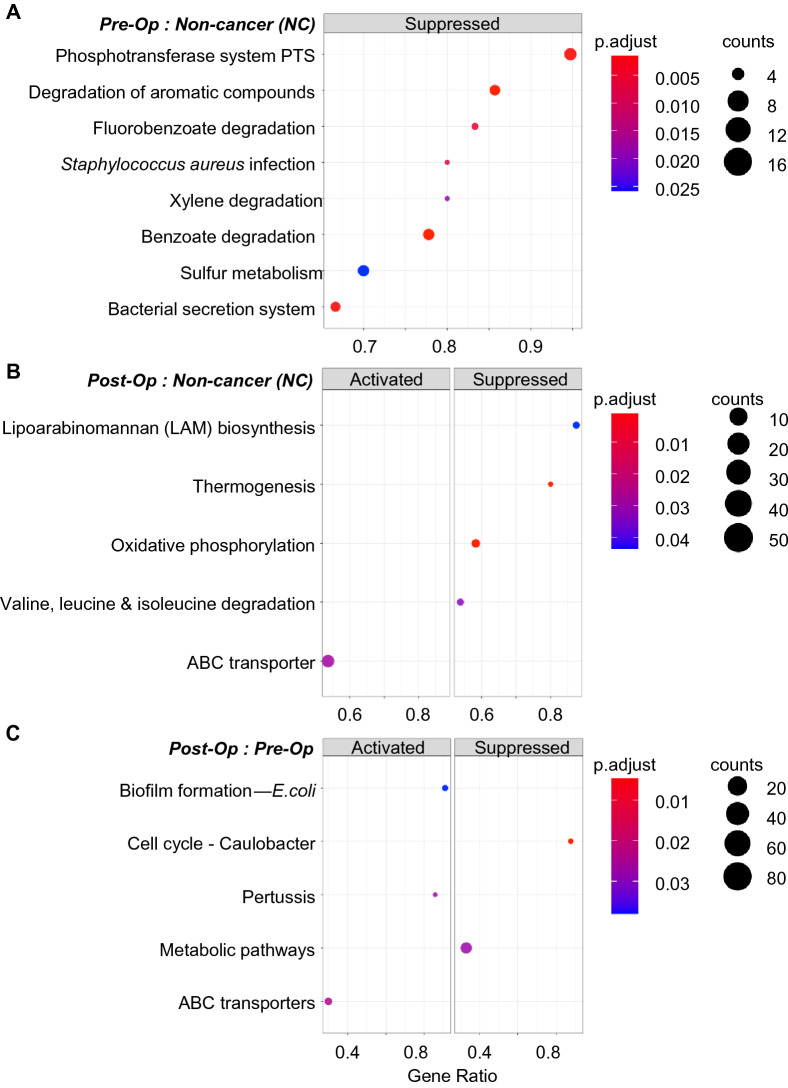
KEGG gene enrichment analysis. (**A**,**B**) Dot plots showing KEGG pathways that were enriched in CRC pre- and post-op compared to NC controls respectively. (**C**) Dot plot shows enriched KEGG pathways in CRC patients pre- and post-op. p. adjust and counts denote Benjamini–Hochberg adjusted p values and gene counts respectively.

As mentioned above, pathway enrichment analysis revealed that “Sulfur metabolism” was significantly lower in pre-op CRC compared to NC controls (Fig. 5A and Supplementary Fig. 2). K00392 is highly associated with pathways including “Sulfur metabolism” and “Microbial metabolism in diverse environments”, indicating a buildup of sulfate in the colonic environment due to the inability to metabolize sulfate to sulfur amino acids^35,36^.

#### Post-op CRC vs NC

When comparing post-op to NC, enrichment analysis showed that Lipoarabinomannan (LAM) biosynthesis was suppressed in post-op compared to NC control group. Lipoarabinomannan is a virulence factor commonly associated with *Mycobacteria tuberculosis* infection. It is also expressed by many other *Actinomycetes* bacteria including *Gordonia, Rhodococcus, Tsukamurella, Amycolatopsis* and *Corynebacterium*. While the sequencing data did not have sufficient resolution to identify each of these Actinomycetes bacteria (*M. tuberculosis* was not detected), we found that the suppression of (LAM) biosynthesis was due to reduced abundance of specific KOs (Supplementary Fig. 2) and ASVs belonging to the Actinomycetaceae family, in particular ASV581 and ASV1102.

Furthermore, KOs related to “Oxidative phosphorylation”, “Thermogenesis” and “Valine, leucine & isoleucine degradation” were suppressed in post-op CRC patients (ɑ = 0.000001) (Fig. 5B and Supplementary Fig. 2). KO K08973 hemJ; protoporphyrinogen IX oxidase was reduced by 4 Log_2_ fold. This KO is highly associated with “Heme biosynthesis, plants and bacteria, glutamate”. Studies have demonstrated that glutamine stimulates the expression of HO-1, a protein that is involved in maintaining intestinal cell proliferation and repair^37^. The presence of pathogenic bacteria such as *Enterobacter spp.*, *Klebsiella spp*., and *Hungatella hathewayi* (Fig. 3B) as well as a compromised immune function suggests that the colonic environment remains in a state of dysbiosis and is prone to damage after surgery.

Interestingly, thermogenesis is suppressed in post-op CRC patients. Studies have reported that amino acids valine, leucine and isoleucine participate in lipogenesis, lipolysis and immune function of the gut^38^. Lipogenesis and lipolysis can be circumvented through supplementation of microbial butyrate. However, butyrate producing bacteria, *Lachnospiraceae*, was observed to have diminished (Fig. 3B) thus subjecting the gut environment to a compromised adaptive thermogenesis^39^. Similarly, reduction in K02274 coxA, ctaD; cytochrome c oxidase subunit I and K02276 coxC, ctaE; cytochrome c oxidase subunit III, which are associated with “Oxidative phosphorylation”, were also observed. Studies have demonstrated that mitochondrial mutations affect downstream processes of the electron transport chain system, which indirectly contribute to an increase in production of reactive oxygen species leading to a compromised repair system^40,41^.

#### Pre- vs post-op CRC

To evaluate the influence of surgery on microbial dynamics, further analysis were carried out by comparing the changes in predicted function profile between pre- and post-op using paired sample test. A total of 25 differentially abundant KOs were identified (BH adjusted P ≤ 0.0015, Supplementary Data 3). These KOs were mapped to KEGG pathways including metabolic pathways (28%); ABC transporters (8%); Carbon metabolism (8%). KOs K19076 cmr2, cas10; CRISPR-associated protein Cmr2, K19141 cmr5; CRISPR-associated protein Cmr5 and K14440 SMARCAL1, HARP were more abundant (3 Log_2_ fold) in pre-op patients compared to NC. These KOs are highly associated with BRITE functions including “Prokaryotic defense system” and “Chromosome and associated proteins” (ɑ < 0.000001) (Fig. 4B, pre-op column, Supplementary Data 2 and 5).

Interestingly, genes associated with PATHWAY function “ABC transporters”, K13890 gsiC; glutathione transport system permease protein and K17315 gtsA; glucose/mannose transport system substrate-binding protein, were found to be over-represented in post-op, suggesting that surgery leads to increase in bacteria that may be responsible for this function in CRC patients (Supplementary Data 3, 4 and 5). Enrichment analysis revealed genes related to “cell cycle—Caulobacter” and “metabolic pathways” were suppressed in post-op CRC patients when compared to their pre-op state (ɑ = 0.001) (Fig. 5C and Supplementary Fig. 2) as demonstrated with a 4 Log_2_ fold reduced expression of K13581 ccrM; modification methylase, K01771 plc; 1-phosphatidylinositol phosphodiesterase, K05304 NANS, SAS; sialic acid synthase and K16649 glft1 (Fig. 4C). As the above-mentioned pathways are related to cellular process and metabolism, their suppression suggests that the cell cycle of the gut microbiome is faulty.

## Discussion

In this pilot study, we compared the gut microbiota of CRC patients (pre- and post-op) with NC individuals. As a pilot study, the number of patients included is small. Therefore, we have taken measures to ensure that only the most reliable data is presented. With a small sample size, only sufficiently large enough effects can be considered genuine. Based on power calculation, results with effect size of 2 would provide 95% confidence with 80% power (see “Results”). Therefore, we have only reported differences that were greater than effect size 2 and at a strict alpha cut-off at ɑ ≤ 0.0001. With these statistical criteria, differences in bacterial composition were identified between CRC patients and NC individuals. The variations in gut microbiota of pre- and post-op patients may suggest possible field change in the “non-diseased” colon that predisposed them to CRC in the first place, due to the findings of persistent microbial dysbiosis after surgery. This study provides insights to alterations in the gut microbiota of post-op CRC patients.

Firstly, we compared the global gut microbiota composition between CRC patients (pre- and post-op) and non-cancer (NC) controls. We found reduction in fecal bacterial species richness and diversity in the CRC patients compared to the NC control group (Fig. 1) which is in line with data published by Cong et al.^42^. However, some studies showed no significant changes in diversity^43–45^. The differences in outcomes may be attributed to different sample sizes or analytical methods. Interestingly, we noticed significant changes in certain microbiota when comparing paired microbial communities between CRC patients pre- and post-op. This may suggest that surgery for CRC not only removes the tumor but is also capable of altering associated gut microbiota communities.

Following which, we analysed the differences in microbiota composition between pre-op, post-op and NC groups. We observed that pre-op CRC group has a significantly higher abundance of several *Bacteroidetes* ASVs and *Odoribacter splanchnicus* compared to NC^19,41^. There is existing literature which suggests microbial dysbiosis having a role in the development of CRC^46–49^. For instance, *Bacteroidetes* and *Odoribacter splanchnicus* are suggested to promote colorectal carcinogenesis. Others have reported association of specific *Bacteroidetes spp.* including *B. caccae* and *B. vulgatus* are known to be involved in destabilizing the colonic wall of the gut, potentially resulting in the progression of CRC^46,47^. At the same time, *Odoribacter* levels is shown to correlate with somatic mutations and cell proliferation resulting in poorer CRC prognosis^50,51^. Interestingly, differences in *Bacteroidetes* ASVs and *Odoribacter splanchnicus* were no longer observed after surgery when compared to NC individuals (Fig. 3A).

At the same time, there was an increased abundance of probiotic bacteria such as *Bifidobacterium *spp. (*Bifidobacterium dentium* and *Bifidobacterium breve*), *Lactobacillus *spp, *Akkermansia muciniphila*, *Anaerostipes caccae* and *Colidextribacter *spp. after surgery (paired analysis between post- and pre-op, Fig. 3B). These probiotics have been demonstrated to fortify the colonic mucus layer, competitively exclude pathogenic bacteria and regulate the colonic environment through regulating of immune cells for anti-tumour activity^52–57^. The increased abundance of beneficial gut genera after surgery such as short-chain fatty acid (SCFA) producing microbiota (*Roseburia spp.* and *Blautia spp.*) and gut barrier enhancer (*Lactobacillus spp.*) suggest that surgery may partially “revert” the gut back to its “NC” pre-cancerous state to a certain degree^30,58^. In addition, presence of pathogenic bacteria such as *Enterobacter *spp., *Klebsiella *spp. and *Hungatella hathewayi* could indicate that the colonic environment is still in a state of dysbiosis as these bacteria are known to be oncogenic^59–61^. Of note, increased *Klebsiella *spp. was also previously reported in post-op compared to pre-op in association with Pertussis (KEGG pathway) enrichment^42^. Microbiota associated with CRC progression such as *Clostridium innocuum*, *Eubacterium brachy*, *Eggerthella *spp. were also observed to be significantly abundant in post-operative CRC patients^40,62,63^. Further, we observed that curative surgery resulted in the reversion of some bacterial strains (i.e., *Bacteroidetes* spp.) to a “non-cancer” level but not others (i.e., *Eubacterium coprostanoligenes* group). On the other hand, certain bacterial groups which were found in equal abundance in NC patients were increased after surgery (i.e., *A. muciniphila*.). This illustrates a possible field-change in microbial composition in the colon of CRC patients, where there is persistence of oncogenic microbial species even after surgery.

Lastly, we examined the functional features inferred from bacterial 16S rRNA gene. Previous studies have shown that metabolic pathways are much more consistent across individuals compared to the differences in bacterial abundance^63^. A healthy gut microbiota may contain specific microbial combinations and essential metabolic pathways that together maintain a two-way beneficial relationship with the host^64^. In our study, we observed differential enrichment of various pathways in patients when compared between the groups. For instance, the inability to metabolise sulfate in the colonic environment was found to be significantly suppressed in pre-op CRC patients compared to NC. In addition, correlation between increased pathogenic bacteria and infectious disease associated pathways (e.g., Pertussis) may be indicative of adverse post-surgery response. Furthermore, correlation of some suppressed pathways to the presence of pathogenic bacteria are indications of how the gut microbiota in CRC patients responded to surgery, thereby influencing its functional stability^40^.

Besides utilising microbial strains which are found in greater abundance in CRC, equal emphasis should be given to other significant strains that are reduced after surgery. The microbial dysbiotic signatures associated with CRC will therefore comprise a specific pattern of alteration in bacteriome. This contrasts with the existing trend of simply identifying specific microbial strains as a complementary tool for the screening and diagnosis of CRC as proposed by various studies. Moreover, these specific patterns can also be used to screen for disease recurrence in CRC patients after surgery, where the return of specific microbial markers can indicate recurrent disease. A follow-up study on the current cohort can seek to examine possible changes in bacteriome in association with disease remission and recurrence.

More importantly, it will be interesting to examine if a similar pattern of microbial dysbiosis is present in patients with adenomatous polyps, which are precursors of CRC, and if these signatures are lost after polypectomy is performed. Studies have reported specific microbes associated with the presence of colonic polyps, which supports the presence of a unique microbial profile even in patients with pre-malignant polyps^65,66^. If found, these microbial dysbiotic signatures may perhaps have the potential to be used as a predictor for patients with polyps and who thus require more urgent colonoscopy. This can help streamline CRC screening and has the potential to guide the allocation and prioritization of colonoscopy, which is costly and not without risks. It may even help guide the indication for surveillance colonoscopy by identifying patients with a greater chance of having recurrent or persistent polyps.

Our study has a number of limitations. Other than the aforementioned small sample size, all recruited CRC patients were diagnosed with left-sided colon cancer. As right and left-sided colon cancers are known to have different molecular biological characteristics and possibly differentially expressed microbial species, the results of our study may not be applicable to right-sided colon cancers^63^. On the other hand, samples from NC group were collected only at a single timepoint and follow up sampling of NC to match the CRC group with exact surgery (e.g. colectomy) was not performed. Although matched NC sampling may provide better comparison with CRC patients, clinical indication for surgery in benign conditions in the NC group is rare (e.g. elective anterior resection for diverticular disease complicated by stricture or fistula). Therefore, the NC controls included are next to ideal for the purpose of this study. They have normal colonoscopy and do not have a history of inflammatory bowel or autoimmune disease (see “Methods”, “Study participants and fecal sample collection”), which could confound the results in this study. Dietary information should also be taken into consideration particularly for CRC patients as changes and differences in diet may influence gut microbiome composition and the risk of CRC^67^. Therefore, inclusion of differences as well as changes in dietary patterns may provide better assessment and identification of confounders when identifying microbial changes before and after surgery in CRC patients.

Specimens used for microbial analysis in this pilot study was based on stool samples, which is postulated to reflect the cancer microbiota due to the downstream shedding of cancer cells. However, it might not fully represent the altered microbial profile of the cancer tissue and we are also unable to study changes in the tumor microenvironment. The bacterial 16S rRNA gene sequencing method, which is a widely employed method of examination of gut microbiome, has technical limitations as 16S rRNA gene amplicon sequencing provides less coverage (absent of other microbes). Unlike shotgun methodology, which can sequence all the genomic material present in the samples, 16S rRNA gene sequencing may not provide a comprehensive assessment of the microbial population with fine resolution at lower taxonomic level. However, both 16S and shotgun methodologies have demonstrated close consensus for bacterial phyla detection^68,69^. We have also analysed our sequencing data using the Dada2 software package to improve the reliability of results. This method was shown to detect fine scale sequence variations with better accuracy and sensitivity compared to other OTU-based output analysis^22,70,71^. The limited resolution provided by 16S rRNA gene sequencing potentially impacted downstream analysis including functional profiling, which can only be “predicated or inferred” using curated pipelines such as Picrust2 employed in this pilot study^23^. Validation with experimental data is important to confirm the changes in functional pathways and their possible influence in the various patient groups. This applies to shotgun sequencing data as well even though all genetic material that encodes for microbial products can potentially be determined by shotgun sequencing. Shotgun results are suitable for downstream bioinformatic analysis to determine the range of functions that can be carried by the organisms. However, the extent of functional activity is not guaranteed regardless of how well the sequencing coverage is, and experimental validation to confirm each of the functional pathways is still required. Therefore, the functional profiles presented in our manuscript should be carefully interpreted since it is an “inferred” data from a small sample population. To note, this study has carefully presented only data that is most reliable based on statistical testing as mentioned above (e.g. effect size > 2 and ɑ ≤ 0.0001).

Although there are limitations in samples size and methodology, our pilot study provides important preliminary insights to the changes of bacterial compositions in CRC with respect to surgical treatment in local Singapore cohort. Our study clearly showed the presence of bacterial composition alteration in NS vs CRC and after surgical intervention. Further validation studies should be carried out in a larger sample size and include both left- and right sided CRC, with a longer duration of follow-up to examine possible predictive microbial signature for disease remission or recurrence. In addition, shotgun sequencing can be employed to assess changes in other microbes including virus and eukaryotes. Further analysis to determine presence of network or co-abundance relationships between different microbial species will help to provide an in-depth evaluation of possible microbial interaction that could influence functional outcomes. The changes in functional outcomes can be measured by metabolomics or lipidomics to assess key microbial and host metabolites such as short chain fatty acid, bile acids, and indole derivatives.

## Conclusion

Microbiome signatures characteristic of CRC likely involves a set of associated microbial alterations which comprise both significant increases and reductions in specific bacterial strains. Elements of this unique microbial dysbiosis of CRC persists even after surgery, suggesting possible field-change in the remnant non-diseased colon. Future studies should involve a larger sample size with shotgun microbiome sequencing data collected at multiple time points after surgery to examine if these dysbiotic patterns truly persist and also correlate with disease outcomes.

## Methods

### Study participants and fecal sample collection

Ethics approval to conduct this study was obtained by the National Healthcare Group domain specific review board (NHG-DSRB), reference number: 2017/01257. All methods were carried out in accordance with relevant guidelines and regulations as outlined by National Healthcare Group domain specific review board, Singapore.

All patients aged 21 and above who were scheduled to undergo elective diagnostic or screening colonoscopy between May 2018 and November 2018 were invited to participate in this study. Informed consent was obtained from all participants and or their legal guardian(s) prior their participation to the study. Participants who were found to have CRC at colonoscopy were assigned to the “Cancer” group, while those who did not have cancer and had normal colonoscopy results were assigned to the “Non-cancer” group. Exclusion criteria were patients who were unable to provide written consent for participation in the study, had pre-existing family history of familial adenomatous polypopsis (FAP) or hereditary non-polypopsis colorectal cancer (HNPCC), diagnosed inflammatory bowel disease, autoimmune diseases and/ or consumption of a prolonged course of antibiotics (> 3 days) during the three months prior to the study or after the surgery.

To compare changes in the gut microbiome profiles of CRC patients before and after surgery, fecal samples at 2 sampling time points were collected from the cancer group. The 2 time points were 1. Prior to colonoscopy (pre-op), before initiation of any cancer treatment, and 2. Six months after surgery (post-op). On the other hand, only one fecal sample prior to colonoscopy was collected from each individual in the Non-cancer group (NC). As there have been reports of immediate microbial alterations after mechanical bowel preparation^20^ which generally recover after 14 days^21^, the stool samples collected prior to colonoscopy for both groups were taken before consumption of polyethylene glycol. A total of 49 fecal samples were collected directly into sterile fecal collection tubes containing RNAlater® (Invitrogen, Lithuania) and 4 sterile glass beads measuring 5 mm in diameter (Merck, Germany). Samples collected by the patients were mailed to the laboratory within one week (± three days). Pre-op samples were collected 24 h (± 12 h) pre-op, and frozen at − 80 °C within 2 h (± 1.5 h) hours, whereas post-op samples were collected at 6 months (± 5 days) post-op by the patients for mailing to the laboratory. All mailed in samples were stored in −80 °C within 24 h (± 12 h) upon receival in the laboratory. Due to the longitudinal nature of this study, all samples were stored at − 80 °C for an average of 6 months (± 2 months) before bulk DNA extraction. All samples are not subjected to any freeze–thaw process before DNA extraction. All DNA extracted were immediately quantified and stored at − 20 °C for 7 days before delivery to external vendors for sequencing. The DNA were packaged in dry ice for delivery to the sequencing vendors (reception by vendors within 24 h).

### Bacterial 16S rRNA gene sequencing

Fecal samples were freshly thawed and vortexed to produce a homogenate and washed once using 1X phosphate buffer saline (Vivantis Technologies, Malaysia). Genomic DNA was extracted using DNeasy PowerSoil Pro Kit (Qiagen, Germany) according to manufacturers’ protocol. The purity and DNA yield for each fecal sample was measured using a nanodrop.

In this study, the V3-V4 region of 16S rRNA was sequenced to analyse composition of the gut microbiota of fecal samples. A PCR targeting the V3-V4 region using forward (5’-CCTACGGGNGGCWGCAG) and reverse primers (5’-GACTACHVGGGTATCTAATCC) with overhang adaptors, were performed as recommended by the 16S metagenomics library kit by Illumina. The quality and quantity of the amplicons were measured using Agilent 4200 TapeStation, picogreen and nanodrop. All samples passed the quality control measurement and proceeded for a second round of PCR step for library preparation. Library qualities were measured using Agilent 4200 TapeStation, picogreen and qPCR. Libraries that passed the quality control measurement were pooled as recommended by Illumina and were sequenced using the MiSeq platform using 2 × 300 PE format.

### Microbiome profiling

Raw fastq files were quality filtered, merged, demultiplexed and denoised using DADA2 (version 1.14.1) microbiome pipeline with default parameters^22^. Output from DADA2, known as amplicon sequence variants (ASVs), were cleaned up by removing chimeras. ASVs is a clustering method for better precision and improve resolution of sequence identity. ASVs were then assigned to their respective taxonomy using Silva (version 138) classifier.

Statistical analysis was carried out in R (version 3.6.3) and analysis of microbiome diversities was carried out using the Phyloseq package (version 1.30.0). Alpha diversity was calculated using Chao1, ACE and Simpson. Diversity differences between CRC (at each sampling time point) and NC groups were analysed using Wilcoxon rank sum test. Inter-group similarities were visualized using principal coordinate analysis (PCoA) of the Bray–Curtis dissimilarity distances. The DeSeq2 analysis package (version 1.26.0—statistical pipeline based on range of distribution) was used was used to identify microbiota that showed significant differential abundances across NC, pre-op and post-op. Read counts/sample and counts across samples were normalised by sample-specific factors determined by median ratio of ASV or KO counts (median of ratio method). The data shown in the figures are the average of the normalised count values, corrected for confounders including gender, race and age of the patients over all samples. For comparisons between pre-Op and post-Op, paired analysis was employed to reflect sampling from the same patient. Predicted functional information from the gut microbiome data was generated using PICRUSt2 (version 2.3.0) pipeline with default parameters^23–25^.

## Supplementary Information

Supplementary Figure 1.

Supplementary Figure 2.

Supplementary Information 1.

Supplementary Information 2.

Supplementary Information 3.

Supplementary Information 4.

Supplementary Information 5.

Supplementary Legends.

## Data Availability

The datasets generated and/or analysed during the current study are available in the NCBI Sequence read archive (SRA) as part of accession BioProject number PRJNA662014.
