# Supplementary figures and images for: Alterations in co-abundant bacteriome in colorectal cancer and its persistence after surgery: a pilot study
Source: Sci Rep. 2022 Jun 14;12:9829. doi: 10.1038/s41598-022-14203-z (PMC9198081; doi:10.1038/s41598-022-14203-z)

## Slide 1
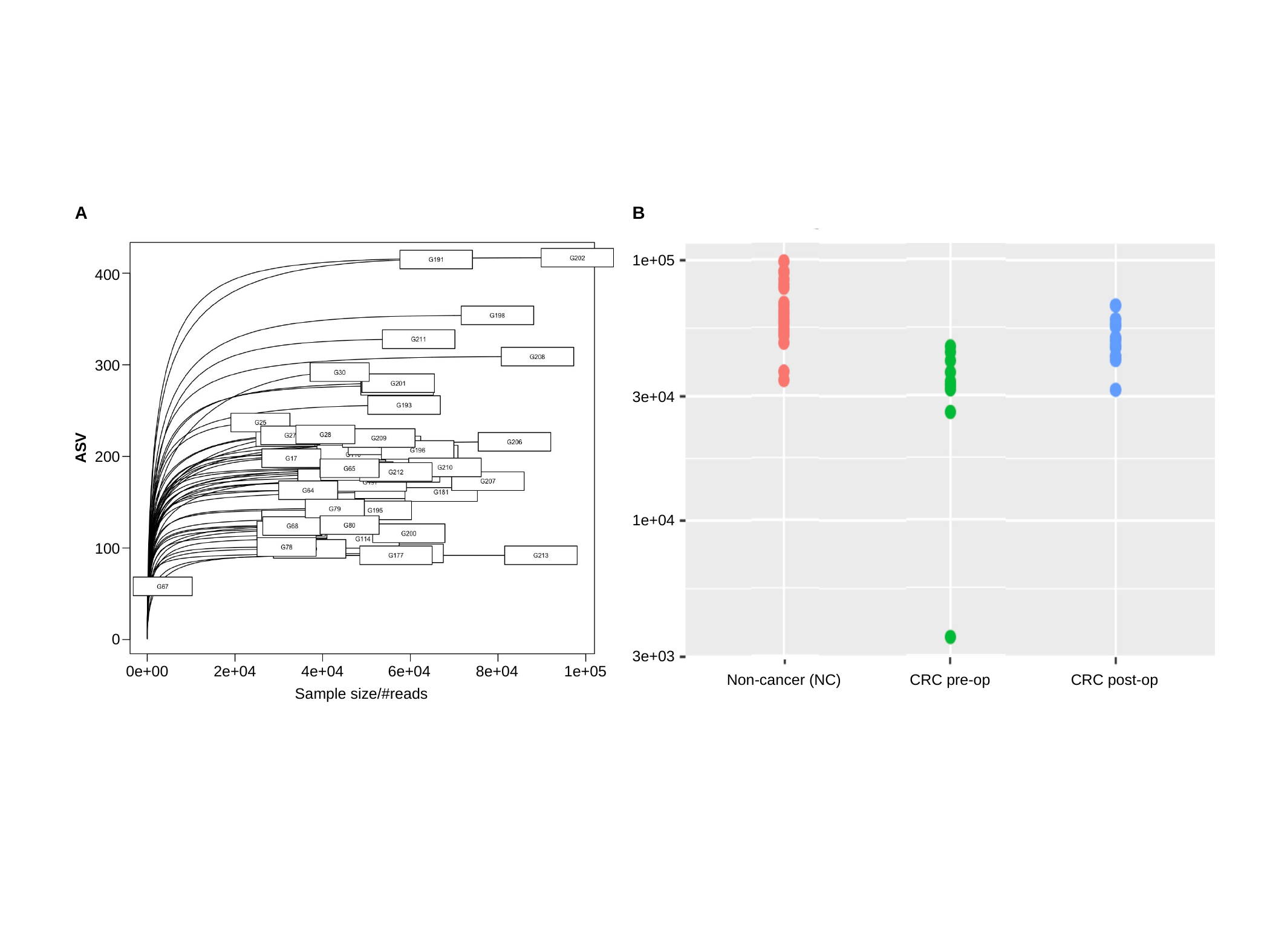

A
B
1e+05
400
300
3e+04
ASV
200
1e+04
100
0
3e+03
0e+00
2e+04
4e+04
6e+04
8e+04
1e+05
Non-cancer (NC)
CRC pre-op
CRC post-op
Sample size/#reads

Supplement: Supplementary file 1 — Supplementary Figure 1. [file 41598_2022_14203_MOESM1_ESM.pptx]

## Slide 1
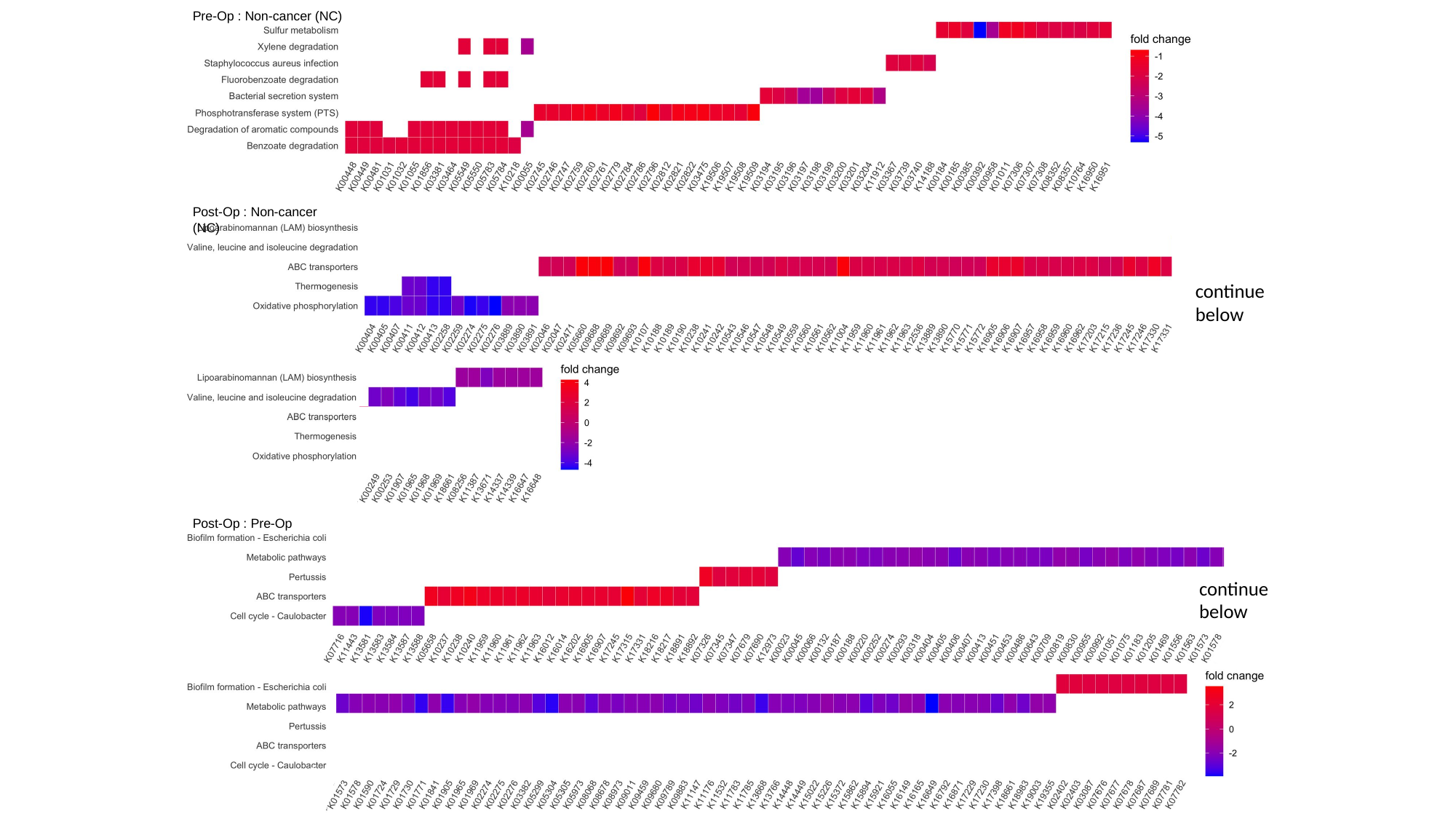

Pre-Op : Non-cancer (NC)
Post-Op : Non-cancer (NC)
continue
below
Post-Op : Pre-Op
continue
below

Supplement: Supplementary file 2 — Supplementary Figure 2. [file 41598_2022_14203_MOESM2_ESM.pptx]
