# Supplementary material for: Alterations in co-abundant bacteriome in colorectal cancer and its persistence after surgery: a pilot study
Source: Sci Rep. 2022 Jun 14;12:9829. doi: 10.1038/s41598-022-14203-z (PMC9198081; doi:10.1038/s41598-022-14203-z)
Supplement: Supplementary file 3 — Supplementary Information 1. [file 41598_2022_14203_MOESM3_ESM.pptx]

## Slide 1
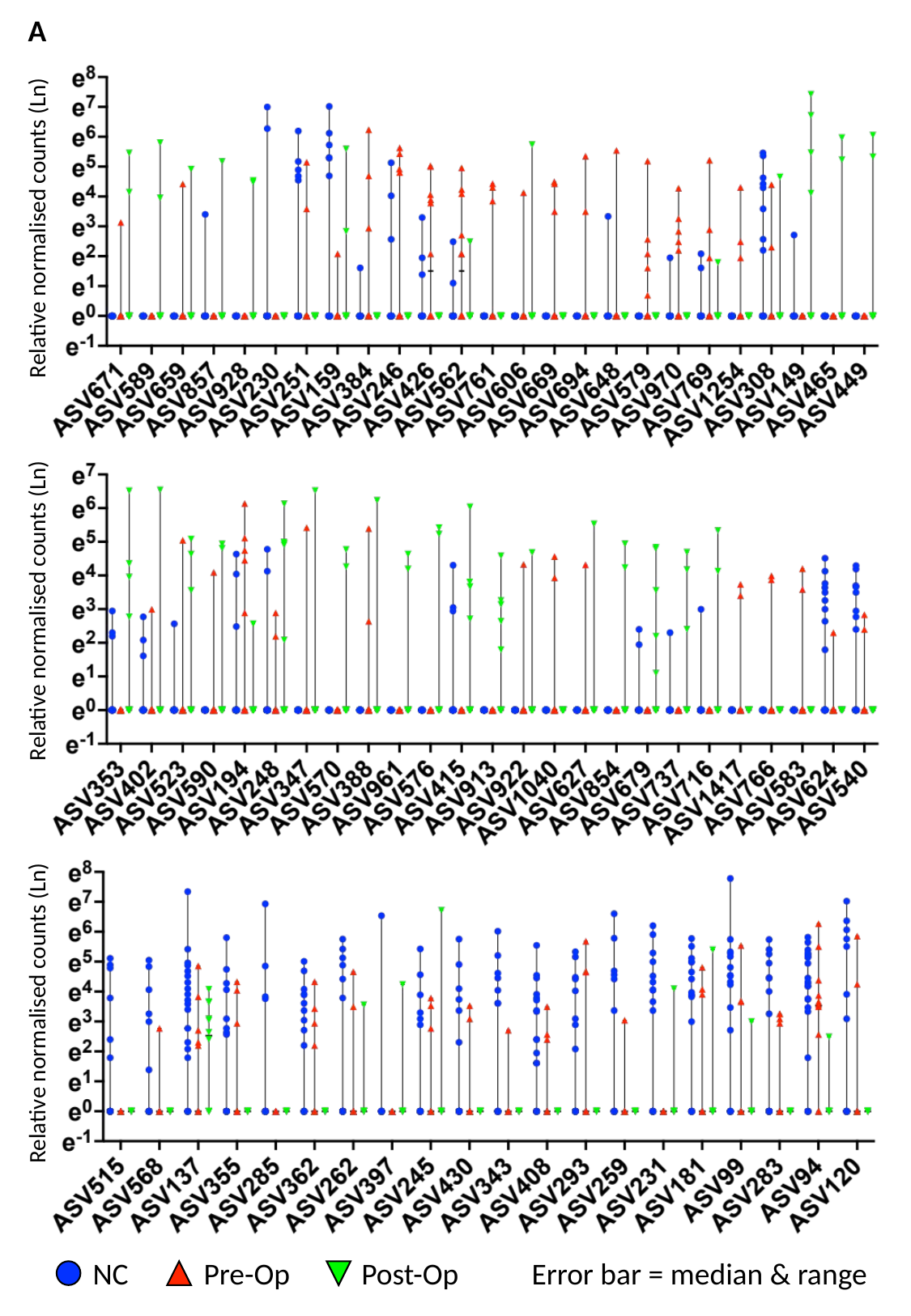

A
Relative normalised counts (Ln)
Relative normalised counts (Ln)
Relative normalised counts (Ln)
NC
Pre-Op
Post-Op
Error bar = median & range

## Slide 2
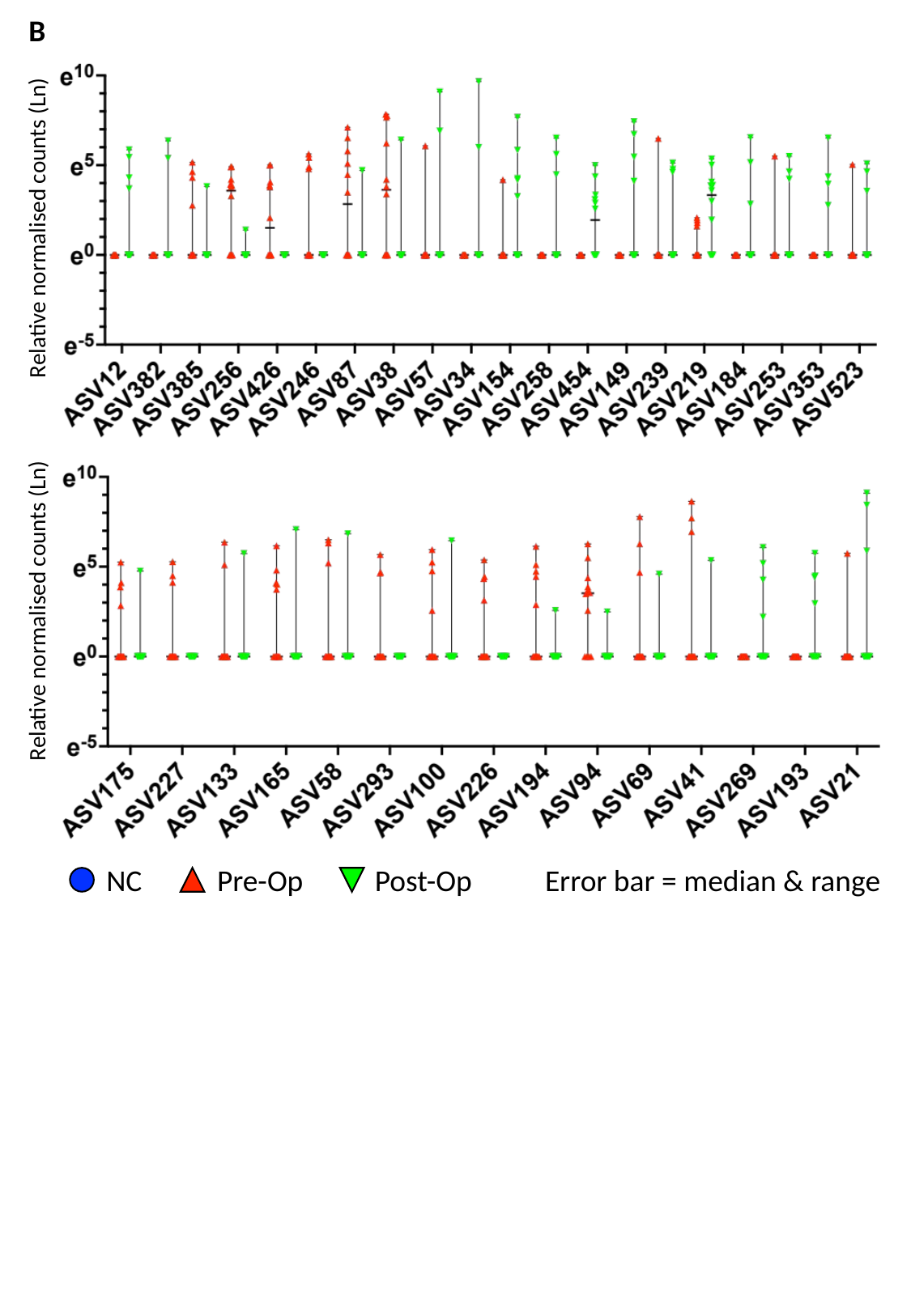

B
Relative normalised counts (Ln)
Relative normalised counts (Ln)
NC
Pre-Op
Post-Op
Error bar = median & range
