# Supplementary material for: Alterations in co-abundant bacteriome in colorectal cancer and its persistence after surgery: a pilot study
Source: Sci Rep. 2022 Jun 14;12:9829. doi: 10.1038/s41598-022-14203-z (PMC9198081; doi:10.1038/s41598-022-14203-z)
Supplement: Supplementary file 8 — Supplementary Legends. [file 41598_2022_14203_MOESM8_ESM.docx]

**Supplementary Figure 2** | Heatmaps showing details of KOs that contributed to the enriched pathways associated with different comparisons.
